# Barriers to the Implementation of Newborn Pulse Oximetry Screening: A Different Perspective

**DOI:** 10.3390/ijns4010004

**Published:** 2018-01-11

**Authors:** Martin Kluckow

**Affiliations:** Department of Neonatal Medicine, Royal North Shore Hospital and University of Sydney, Sydney, NSW 2065, Australia; martin.kluckow@sydney.edu.au; Tel.: +61-2-9463-2180

**Keywords:** pulse oximetry, neonate, congenital heart disease, screening

## Abstract

Pulse oximetry screening of the well newborn to assist in the diagnosis of critical congenital heart disease (CCHD) is increasingly being adopted. There are advantages to diagnosing CCHD prior to collapse, particularly if this occurs outside of the hospital setting. The current recommended approach links pulse oximetry screening with the assessment for CCHD. An alternative approach is to document the oxygen saturation as part of a routine set of vital signs in each newborn infant prior to discharge, delinking the measurement of oxygen saturation from assessment for CCHD. This approach, the way that many hospitals which contribute to the Australian New Zealand Neonatal Network (ANZNN) have introduced screening, has the potential benefits of decreasing parental anxiety and expectation, not requiring specific consent, changing the interpretation of false positives and therefore the timing of the test, and removing the pressure to perform an immediate echocardiogram if the test is positive. There are advantages of introducing a formal screening program, including the attainment of adequate funding and a universal approach, but the barriers noted above need to be dealt with and the process of acceptance by a national body as a screening test can take many years.

## 1. Introduction

Reviews suggest that about 30% of infants with critical congenital heart disease (CCHD) leave hospital undiagnosed and that, in cardiovascular deaths occurring within the first week of life, the malformation was not identified before death in one out of four [1,2]. Neurological outcome is related to the presentation of the disease, with infants who collapse prior to presentation having a significantly worse outcome than those that are identified prior to collapse [3]. There is therefore a need for the development of effective screening tests for CCHD. Current screening for congenital heart defects has relied on a mid-trimester ultrasound scan, which is operator-dependent and at present detects <50% of CHD and about 60% of CCHD requiring surgery in the first month of life [4,5]. In Sweden, 26% of newborns with CCHD were sent home without being diagnosed [6].

Pulse oximetry has been evaluated in multiple studies as a screening test for CCHD. A high sensitivity is clearly important where a test is used to screen for a serious but treatable disease. Ewer et al. [7] in a test accuracy study showed that pulse oximetry had a sensitivity of 58% for critical (likely to require treatment in the first month) and 29% for all major (likely to require treatment in the first year) lesions when antenatal screening was negative. A systematic review and meta-analysis by Thangaratinam et al. [8] including 13 studies and almost 230,000 babies showed the overall sensitivity of pulse oximetry for the detection of critical congenital heart defects was 76.5%. In this review, there were no significant differences in sensitivity for pulse oximetry in the foot alone versus in both foot and right hand. The specificity was 99.9%, with an overall false-positive rate of 0.14%. The equipment is readily available and does not require calibration; the monitoring is minimally invasive and familiar to most parents and staff. Despite all of these potential screening advantages, the uptake of pulse oximetry screening for CCHD has not been universal. This paper aims to identify and review the barriers to the implementation of pulse oximetry as a screening test for CCHD.

## 2. Australian/New Zealand Progress

The adoption of pulse oximetry for screening for critical congenital heart disease has progressed substantially around the world, led by the development and adoption of screening guidelines in North America by the American Academy of Pediatrics (AAP) in 2011 [9]. The adoption of pulse oximetry screening in Australia/New Zealand has been on a hospital-by-hospital, state-by-state basis. New Zealand has recently proposed a countrywide adoption of screening at all health care facility levels and is currently exploring the feasibility of this [10]. A recent survey of all of the Australian/New Zealand Neonatal Intensive Care Units (Unpublished 2017) concluded that 77% of all units have implemented a screening program. Three units in New Zealand were not screening pending the introduction of a National screening program. Two units in Australia had suspended their screening programs due to resourcing implications both at the primary screen and in dealing with positive test results. Most units have adopted a screening guideline similar to either the AAP-recommended one or one based on the PulseOx study [7], but with some practical differences, particularly in terms of the timing of the screen and response to a positive screen. None of the units required a mandatory echocardiogram as part of the response to a positive screen.

The approach in Australia has been driven in part by some modification of the basic tenants of pulse oximetry screening for CCHD. Whilst the focus in the USA and the UK has been on the implementation of a formal screening program for CCHD, the discussion in Australia and New Zealand has been on the use of the terminology of “Pulse oximetry screening for critical congenital heart disease” versus “Pulse oximetry screening of the well newborn”, the timing of pulse oximetry screening, the interpretation and significance of false positives, and the appropriate action for babies who screen positive, all of which have been areas of controversy during the implementation of universal routine pulse oximetry screening in many countries, including the United Kingdom [11].

## 3. Challenges in Introducing Pulse Oximetry Screening for CCHD

### 3.1. Screening for CCHD or Documentation of a Vital Sign

Pulse oximetry is used routinely in the assessment of adult patients admitted to hospital. Early warning scores have been developed, inclusive of routine saturation checks, to identify patients before clinical deterioration and preventing admissions to the intensive care unit. Saturation documentation forms part of Paediatric early warning systems, such as the Cardiff and Vale Paediatric Early Warning System and the Melbourne criterion for activation of medical emergency teams [12]. In Australia, local state health authorities have implemented programs such as “Between the Flags” to recognise and respond to patients when their clinical condition starts to deteriorate, which include documenting oxygen saturation [13]. Saturation monitoring has been proposed as an adjunct to the assessment of the newborn in the delivery room and as a routine vital sign assessment [14]. It is proposed that the documentation of oxygen saturation in the newborn should be an integral part of normal vital sign documentation, equivalent in importance to pulse, respirations, heart rate, and blood pressure. Introducing pulse oximetry as part of a routine observational assessment changes the emphasis of a pulse oximetry measure from screening for CCHD (still achieved) to documentation of the fifth vital sign [15]. As a result, it has been our observation that many of the barriers to CCHD screening are minimized, including parental anxiety about the link with CCHD and subsequent refusal of the screen [16], the need to obtain consent in some programs, which can be threatening to parents necessitating an opt-out clause in some countries, including the USA [17], the concept of a false positive for CCHD when the infant has a positive pulse oximetry screen (i.e., is noted to be hypoxic) but is not diagnosed with CCHD, and finally the response to a positive screen, which does not have to be a mandated echocardiogram with all of its resource implications [18].

### 3.2. Linking ‘Pulse Oximetry Screening’ to ‘Screening for CCHD’

Referring to the screening program as “Pulse oximetry screening of the well newborn” rather than a “Program to screen for critical congenital heart disease” has resulted in better acceptance of the screening program for clinicians and parents in our setting [18]. Pulse oximetry screening identifies some forms of cyanotic heart disease, but does not screen for all CCHD. Some babies with CCHD are missed using pulse oximetry screening, particularly those with obstruction of the aorta. There is a risk of false parental reassurance of absence of congenital heart diseases with the use of the term ‘Pulse oximetry screening for CCHD’.

The terminology “Screening for CCHD” may raise anxiety, as it introduces the possibility of a child having a serious health condition. In a recent article by Powell et al. [16] evaluating the acceptability of pulse oximetry screening to mothers, white British and Irish mothers had the lowest rate of decline (5%), while all other minor ethnic groups had an increased likelihood of declining the screening in a research setting (up to 21% in African women). Post-hoc analysis indicated that participants of minor ethnic origin were more anxious, more depressed, less satisfied, and more stressed than the white population who participated in the study. In our opinion, replacing the terminology with “routine pulse oximetry screening” as a documentation of a vital sign undertaken on all babies born in hospital is less likely to raise unnecessary anxiety in parents. The interesting requirement for an opt-out clause in the pulse oximetry screening program in the United States [17] is likely to have resulted from similar observations of parental anxiety.

### 3.3. Timing of Pulse Oximetry Screening and Significance of False Positives

The AAP work group recommends that screening should not begin until after 24 h of life, or as late as possible if an earlier discharge is planned, and be completed on the second day of life. Dawson et al. [19] have defined reference data for oxygen saturation in healthy full-term infants during their first 24 h of life. The time to reach a stable saturation >95% is generally 20 min in healthy babies (range 3–90 min), so waiting for 24 h is cautious. Earlier screening can lead to more false-positive results because of the transition from fetal to neonatal circulation and the stabilization of systemic oxygen saturation levels [9]. Thangaratinam et al. [8] showed that the false-positive rate for detection of CCHD was particularly low when newborn pulse oximetry was done after 24 h from birth than when it was done before 24 h: 0.05% versus 0.50%. Consequently, many screening programs have chosen to screen after 24 h to decrease the false positives for CCHD. An alternative way of looking at this is that the infants picked up on a positive screening test are infants with low oxygen saturation, regardless of the aetiology, and that any infant with low saturation requires investigation. When the population of infants with a false positive for CCHD are reviewed in the large data sets of screening, more than 50% of them will have important pathology, including congenital pneumonia, sepsis, meconium aspiration syndrome, milder forms of congenital heart disease, and failure to transition (eg. persistent pulmonary hypertension of the newborn (PPHN), transient tachypnea of the newborn (TTN)) [7,20,21,22]. Although these studies were not specifically designed to assess the cohort of false positives, a false positive result suggests a ‘hypoxic’ baby and a baby with undiagnosed Group B streptococcal sepsis, pneumonia, or PPHN is just as likely to collapse and die as a baby with undiagnosed CCHD. If documentation of saturation is agreed to be a routine vital sign, are we delaying the documentation of saturation in our babies for the wrong reasons?

When combined with the routine anomaly scan and newborn physical examination, early (4–24 h) pulse oximetry screening adds value to existing screening procedures and is likely to be useful for the identification of cases of CCHD that would otherwise go undetected. The added value in pulse oximetry screening over and above physical examination has been quantitated in two studies. deWahl Granelli [23] showed an increase in sensitivity of CCHD detection from 63% to 83% with specificity remaining at 98%. Similarly, Zhao et al. showed increased sensitivity of CCHD detection from 77.4% to 93.2% with the addition of pulse oximetry screening to the newborn examination [24].

There is clear data to show that infants with CCHD who present collapsed will have a worse neurological outcome than those who are identified before a collapse [3]. As a significant number of infants with CCHD present in the first 24 h with early ductal closure [25], planning a screening program in the first 24 h will result in less collapsed presentations and provide an opportunity for earlier stabilization and intervention. An added benefit is that screening within the first 24 h is less likely to interfere with the discharge process, particularly in those false positive cases that require only minimal intervention, such as a period of observation. The pros and cons of early versus late screening are presented in Table 1.

### 3.4. Response to a Positive Screen

The number of false positives for CCHD arising from the physical examination is significantly more than that from pulse oximetry screening [24]. One of the perceived impediments in introducing a pulse oximetry screening program is the need for rapid access to cardiology services to perform an echocardiogram in the event of a failed screening test. In reality, these are babies likely to present to health care providers at some point, apart from the small number with transitional problems that will self-resolve. All health care facilities managing deliveries and newborn babies should already have existing referral and escalation pathways to deal with infants with suspected CHD. Pulse oximetry screening is simply a complement to the existing mechanisms whereby suspected CHD may be identified on physical examination in response to a member of staff reporting a ‘dusky’ baby or after a low saturation measure during an ad hoc pulse oximetry measure in a dusky appearing baby. These presentations are no different to a ‘positive’ pulse oximetry screen. In the published pulse oximetry studies, all babies with failed screens were referred for an echocardiogram to allow for full ascertainment of sensitivity and specificity in those babies with a low pulse oximetry reading. In fact, the AAP working group recommended that any newborn with a positive screen result first requires a comprehensive evaluation for causes of hypoxemia. In the absence of other findings to explain hypoxemia, CCHD needs to be excluded on the basis of a diagnostic echocardiogram (which would involve an echocardiogram within the hospital or birthing center or transport to another institution) [9]. The need for an echocardiogram should be determined on a case-by-case basis as it would be for other presentations of potential congenital heart disease (murmur, visible cyanosis). The actual number of infants requiring further investigation as a result of a failed pulse oximetry can be surprisingly small, and in particular the requirement for extra echocardiograms is minimal [18].

## 4. Pulse Oximetry of the Well Newborn versus Screening for CCHD

**Figure 1:**
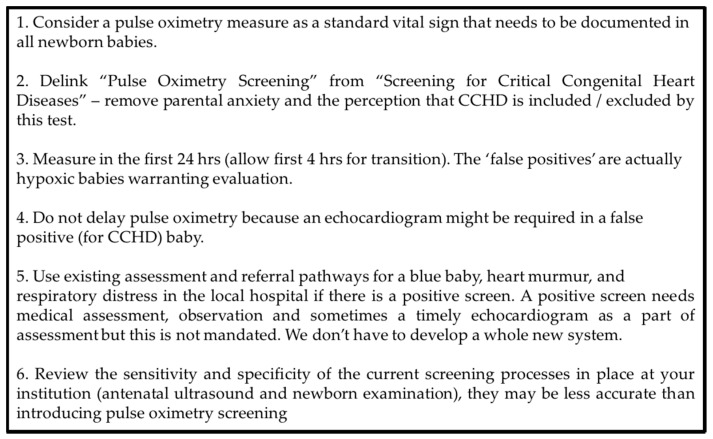
Pulse oximetry screening: A new paradigm.

The dilemma that many countries are facing when introducing a program to identify hypoxic/borderline hypoxic infants is whether to mandate this as part of a formal national screening program or to introduce pulse oximetry as part of the routine observations performed on a newborn infant. There are pros and cons of each approach and these are summarized in Table 2. The introduction of pulse oximetry for all well newborns prior to discharge, by documenting SpO2 as the 5th vital sign, is appealing and relatively straight forward and the equipment and skills to measure it are already generally available. It is our opinion that delinking the term CCHD from the test allows for the documentation of SpO2 without needing to explain in detail about CCHD and complications that might occur from this, resulting in decreased parental anxiety, a reduced possibility of misinterpretation that CHD has been completely ruled out, false positives becoming less relevant such that earlier screening can be proposed, and a less likely implication of the need for a mandated echocardiogram in the event of a failed screen (Figure 1).

Importantly, the detailed requirements needed to satisfy inclusion as a formal country-wide screening test are not needed: these requirements can result in significant delays in the introduction of a screening program. The downside of this approach is that there may not be true nationwide screening, particularly at smaller, under-resourced hospitals. The approach may result in a less-uniform approach and lack of a formalized collection of results to understand the impact of screening. In contrast, a formal application to include pulse oximetry screening for CCHD as a part of a country screening program results in proper resourcing, oversight, and governance. It is more likely that all babies at all levels will be screened. However, the process takes time (5 years and still proceeding in the case of the United Kingdom) and will still suffer from all of the issues discussed above when pulse oximetry measures are linked to screening for CCHD. The Nordic countries have been successful in the approach of a hospital-by-hospital introduction of screening, resulting in an overall coverage of screening of close to 100% [26].

## 5. Conclusions

Currently in our part of the world, Australia has chosen to follow the introduction of screening on a hospital-by-hospital basis, adopting many of the tenants of the 5th vital sign approach, whilst New Zealand has signaled its intention to adopt a country-wide screening program due to some of the unique challenges of health care delivery they have [10]. It will be interesting to track how each country achieves the common aim of improving detection of CCHD and thus reducing deaths and neurodevelopmental injury associated with these significant congenital abnormalities. The body of research to date strongly supports the utility of screening all well newborn infants with pulse oximetry. However, the implementation of screening as performed in the research framework into the real life scenario has been impeded by many of the issues discussed in this paper. As more Units and countries describe their approach to screening and outcomes, a more balanced approach to the introduction of pulse oximetry screening is likely to be achieved.

## Figures and Tables

**Table 1:** Pros and cons of screening before and after 24 h of age.

<24 h of Age	>24 h of Age
Increased detection of significant and major CHD	Increased detection of significant and major CHD
Optimal for prevention of postnatal hypoxia	Not optimal but still prevents some hypoxic events
Higher false positive rate for CCHD (0.5%)	Lower false positive rate for CCHD (0.05%)
Detection of other pathology (up to 50% of all false positives)	Detection of other pathology (up to 50% of all false positives)
Often still in hospital: doesn’t disrupt discharge process	May disrupt discharge process

CHD: congenital heart disease; CCHD: critical congenital heart disease.

**Table 2:** Formalised screening program versus vital sign documentation.

Screening Program	Hospital Led/5th Vital Sign
Meeting screening test criteria, Competing with other national screening programs for funding	More easily achievable without a complex application process
Research based: almost 500,000 babies tested	Harder to justify as not linked to CCHD research
Country-wide introduction, mandated, uniformity of coverage	Gaps in provision, Ad Hoc screening
Properly resourced and funded. Quality improvement more easily achieved	Resourcing is not excessive so achievable by most hospitals
CHD is a tested hard outcome	Importance of other diagnoses and timing of the test
Follows existing research based algorithms: reduced flexibility	Delink from CCHD terminology: reduces pressure from false positives and need for echocardiogram.
